# PaCS-Toolkit: Optimized Software Utilities for Parallel Cascade Selection Molecular Dynamics (PaCS-MD) Simulations and Subsequent Analyses

**DOI:** 10.1021/acs.jpcb.4c01271

**Published:** 2024-04-05

**Authors:** Shinji Ikizawa, Tatsuki Hori, Tegar Nurwahyu Wijaya, Hiroshi Kono, Zhen Bai, Tatsuhiro Kimizono, Wenbo Lu, Duy Phuoc Tran, Akio Kitao

**Affiliations:** † School of Life Science and Technology, Tokyo Institute of Technology, 2-12-2 Ookayama, Meguro, Tokyo 152-8550, Japan; ‡ Department of Chemistry, Universitas Pertamina, Jl. Teuku Nyak Arief, Simprug, Jakarta 12220, Indonesia

## Abstract

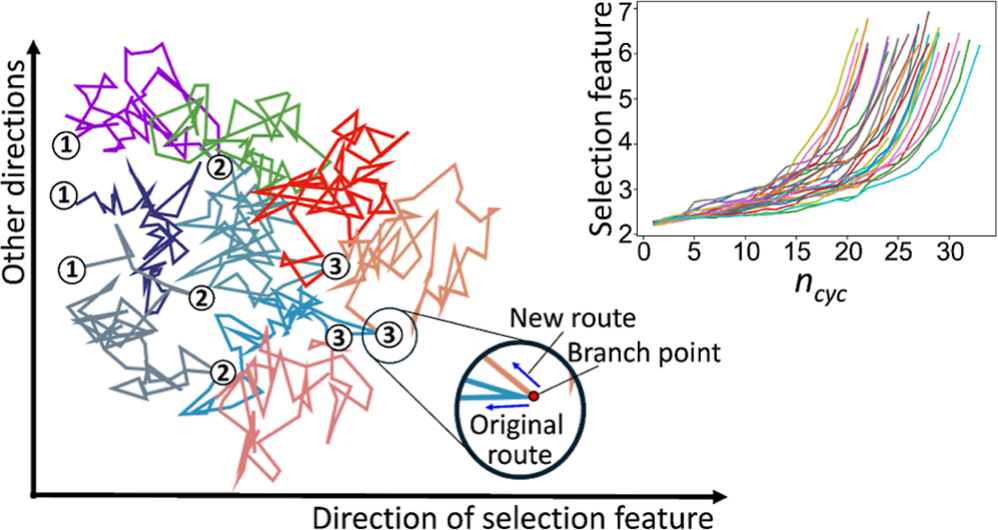

Parallel cascade selection molecular dynamics (PaCS-MD) is an enhanced conformational sampling method conducted as a “repetition of time leaps in parallel worlds”, comprising cycles of multiple molecular dynamics (MD) simulations performed in parallel and selection of the initial structures of MDs for the next cycle. We developed PaCS-Toolkit, an optimized software utility enabling the use of different MD software and trajectory analysis tools to facilitate the execution of the PaCS-MD simulation and analyze the obtained trajectories, including the preparation for the subsequent construction of the Markov state model. PaCS-Toolkit is coded with Python, is compatible with various computing environments, and allows for easy customization by editing the configuration file and specifying the MD software and analysis tools to be used. We present the software design of PaCS-Toolkit and demonstrate applications of PaCS-MD variations: original targeted PaCS-MD to peptide folding; rmsdPaCS-MD to protein domain motion; and dissociation PaCS-MD to ligand dissociation from adenosine A_2A_ receptor.

## 1 Introduction

Parallel cascade selection molecular dynamics (PaCS-MD) is an enhanced conformational sampling method based on molecular dynamics (MD) simulation using only standard force fields, and consists of cycles of multiple MD simulations conducted in parallel using different conditions (replicas) and selections of the initial structures closest to a target for the next cycle.^1−14^ This procedure can be considered as a “repetition of time leaps in parallel worlds” until reaching a target, which enables complete parallel execution of all the MD simulations for each cycle or computation with distributed computing. PaCS-MD has been shown to be very efficient in sampling large protein conformational change,^1,2,6,8−10,13,15^ peptide folding,^1^ the dissociation and association of protein/ligand, protein/peptide, and protein/DNA complexes^4,5,7,11,13,14,16^ without applying any bias during each MD simulation.

**Figure 1:**
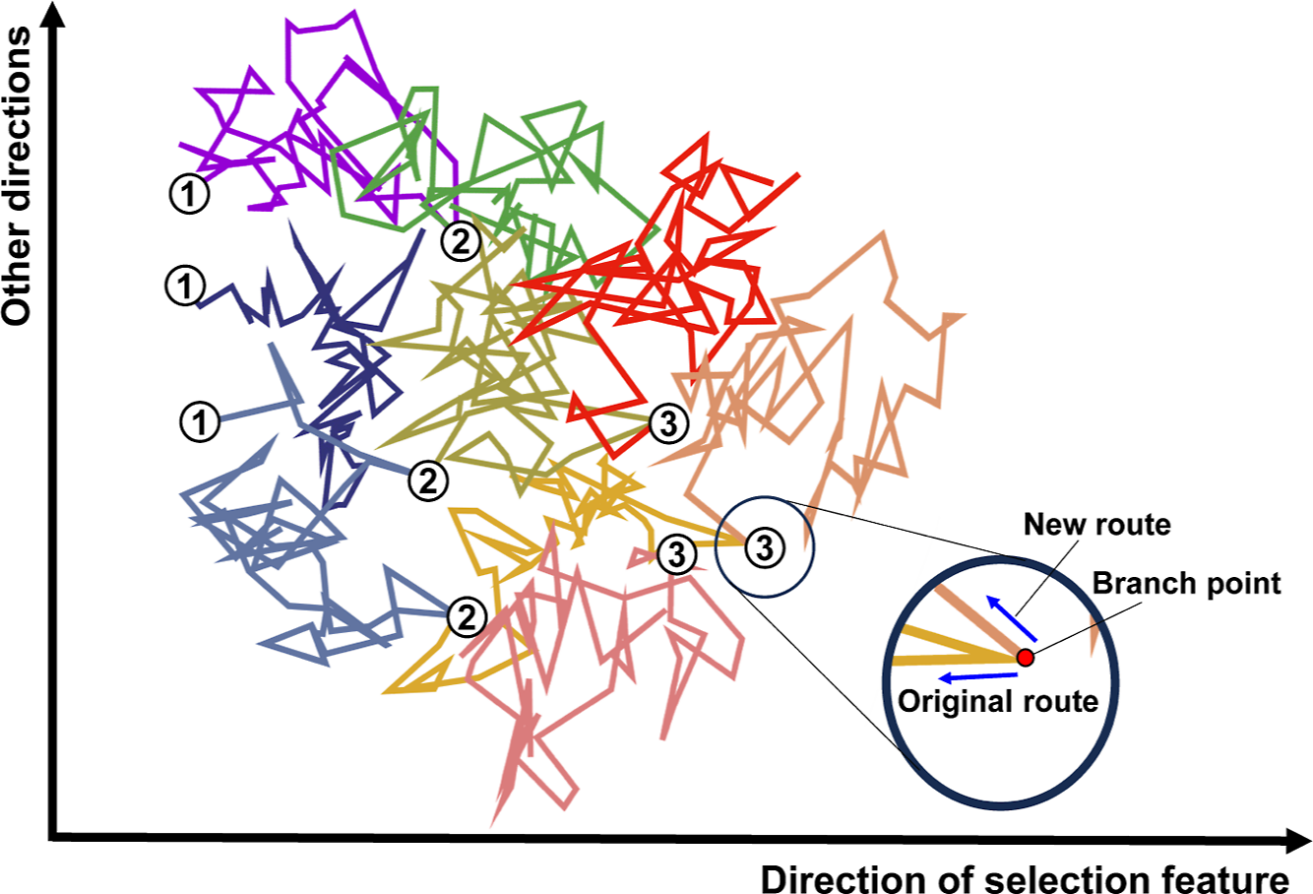
Schematic illustration of how PaCS-MD trajectories evolve during the cycles, shown as projections onto the space spanned by the direction of the selection feature and other directions for the case of *n*_rep_ = 3. The circled numbers represent cycle numbers and are marked with the positions of the initial structures of each cycle.

Typical enhanced sampling simulations with additional bias may require the modification of MD software to add a new function to calculate the bias or to adjust bias parameters to avoid applying too much perturbation to the system. In contrast, PaCS-MD does not require software modifications or such parameter adjustments.^4^ The sampling efficiency along a certain direction in PaCS-MD is enhanced by introducing a quantity for the selection (“selection feature” hereafter). MD snapshots generated in each cycle are then rank ordered based on the selection feature, and the top *n*_rep_ snapshots (*n*_rep_: the number of replicas simulated in each cycle) are employed as the initial structures for the next cycle (Figure 1). This selection protocol effectively harvests snapshots from the edge of distributions along the direction of the selection feature, which are rarely reached on the MD time scale. Therefore, the selection considerably increases the probabilities of relatively rare events occurring in each cycle, thus greatly enhancing conformational sampling along the selection feature after the accumulation of many cycles. The selection protocol generates a set of MD trajectories evolved in a cascaded manner (see Figure 4 of ref (1)).

In addition, velocities are typically reinitialized at the beginning of each cycle based on their Maxwell–Boltzmann distribution, so the trajectories in the new cycle are significantly modulated and new routes are explored from the branch points compared to those in the previous cycle (Figure 1). Significant benefits of the reinitializing protocol have been recognized in the late 1990s in the case of short (120 ps) MD simulations of a protein, in which considerable differences along dominant principal components are induced only by the initial velocity variations.^17^ The efficiency of the reinitializing protocol in PaCS-MD was also revisited and reconfirmed recently.^18^ The ratio of acceleration estimated as the experimentally observed time scale of the event divided by the simulation time of PaCS-MD can be 10^8^ or higher. For example, the actual time scale of dissociation of a protein/peptide complex deduced from dissociation rate constants is on the order of seconds, whereas the simulation time of PaCS-MD is nanoseconds to tens of nanoseconds.^5,7^

The choice of the selection feature is essential for enhancing sampling efficiency in PaCS-MD and a variety of selection protocols have been proposed.^1−10,13,14^ This is relatively straightforward when the target directions along which conformational sampling will be enhanced are identified in advance. The original version of PaCS-MD, sometimes called targeted PaCS-MD or t-PaCS-MD, selects the MD snapshots closest to the target structure in the root-mean-square deviation (RMSD) of the atoms. This approach can efficiently generate pathways for protein conformational transitions and for peptide folding.^1^ In contrast to t-PaCS-MD, rmsdPaCS-MD moves the system the farthest in RMSD from the initial structure.^13^ The distance between two regions in a protein, such as an interdomain distance^6^ or interlid distance,^15^ are used as selection features to enhance the open-close motions of proteins. The intercenter-of-mass distance between two molecules (*d*_com_)^4,5,7,11,19^ is also used as a selection feature to enhance intermolecular dynamics. Dissociation PaCS-MD (dPaCS-MD), in which the snapshots with the longest *d*_com_ are selected, can generate dissociation pathways from complexes, e.g., protein/ligand,^4,11^ protein/peptide,^5,19^ and protein/DNA^16^ complexes within a simulation time of a few to tens of nanoseconds. Association and dissociation PaCS-MD (a/dPaCS-MD) consists of switching between the cycles of association PaCS-MD (aPaCS-MD) conducted by selecting the closest snapshots in intermolecular *d*_com_ and the cycles of dPaCS-MD. This procedure generated many encounter complexes between MDM2 protein and an intrinsically disordered region of p53 protein. Subsequent additional relaxation MD simulations started from many a/dPaCS-MD-generated encounter complexes and Markov state model (MSM) analysis provided the global free energy minimum structure as the conformation closest and very similar to the crystal complex structure among the sampled conformation.^7^ LB-PaCS-MD is simulated in a ligand-concentrated environment and selects the closest *d*_com_ snapshots for frequent sampling of binding pathways.^14^ PaCS-Fit expands the concept of PaCS-MD to the use of experimental data, using better fitting to low-resolution structure data as the selection feature and constructing structural models based on small-angle X-ray scattering and cryo-electron microscopy data.^20^

Variations of PaCS-MD enhance sampling, even when specific target directions are unidentified. The first version of this approach is nontargeted PaCS-MD (nt-PaCS-MD),^2^ in which structures significantly deviating from an average are selected based on Gram–Schmidt orthogonalization of the distribution of the sampled snapshots. Edge expansion PaCS-MD (eePaCS-MD)^8,9^ selects the edges and vertices of the already sampled conformational space in a multidimensional principal component subspace by solving the “convex hull problem”.^21^ The approach conducted in combination with accelerated MD^22^ and is called eePaCS-aMD further improving the efficiency of sampling. Anomaly detection PaCS-MD (ad-PaCS-MD)^10^ employs an anomaly detection generative adversarial network (anoGAN)^23^ for the selection process. Independent nontargeted PaCS-MD (Ino-PaCS-MD) is an extension of nt-PaCS-MD, wherein multiple nt-PaCS-MD are started from different initial configurations.^12^ Tree search MD (TS-MD)^24^ employs a reinforcement learning algorithm, called upper bounds for trees (UCT),^25^ for selection. Harada and co-workers developed many simulation methods comprising cycles of parallel MDs and selections of the initial structures, and proposed them with different names, e.g., fluctuation flooding method (FFM),^26^ outlier folding (OFLOOD),^27−29^ taboo search (TBSA),^30,31^ structural dissimilarity sampling (SDS),^32^ extended SDS,^33^ and self-avoiding conformational sampling (SACS).^34^

PaCS-MD shares similar features with weighted ensemble (WE)^35−41^ and forward flux sampling.^42,43^ PaCS-MD spreads trajectories using the aforementioned cascading procedure, whereas WE is conducted based on the “splitting and merging” of trajectories.^38−41^ Due to differences between these approaches, trajectory weights in WE can be obtained with the established procedures, whereas PaCS-MD typically requires different post processing methods. Early PaCS-MD development used the generated trajectories as starting structures for subsequent sampling simulations, such as umbrella sampling as shown in previous examples,^1,4^ although recently the Markov state model (MSM)^44−46^ is more frequently used for further analysis. The velocity reinitialization protocol introduces disconnectivity among a set of PaCS-MD-generated trajectories in phase space, but trajectories are mutually connected in conformational space due to the branching of the trajectories. MSM that assumes a Markov process is thus a suitable analysis method for PaCS-MD-generated trajectories. The PaCS-MD/MSM combination is widely used to calculate the free energy landscape of conformational changes,^6,8,9,13,15^ standard binding free energy (Δ*G*°),^4,5,7,11,13,16,19^ the association (*k*_on_) and dissociation rate constants (*k*_off_) of protein complexes,^5,7^ and flux along conformational transition pathways.^7,15^ If sampling is insufficient to construct an MSM, additional MD simulations can be added afterward.^11,19^ In the MSM step, the sampled snapshots are grouped into discrete microstates based on certain features (termed MSM features hereafter), which are typically defined as collective coordinates that well characterize matters of interest. PaCS-MD accelerates dynamics along the selection feature but less so dynamics along other directions. Noteworthily, the MSM features can be different from the selection feature and can be decided after PaCS-MD. One trial of PaCS-MD samples the conformational space along a relatively narrow pathway.^4,5^ Therefore, multiple trials of PaCS-MD should be performed to cover a broader conformational space. This process is also important to obtain more statistics, especially when users plan to use MSM for post PaCS-MD analysis. MD simulations in PaCS-MD are typically conducted without bias, but a set of generated MD trajectories might contain artifacts introduced by the selection. MSM analysis likely reduces the effects of potential biases on the set of PaCS-MD trajectories.

Since PaCS-MD is conducted as a combination of MD simulations using only standard force fields without extra bias, it can be performed without the need to modify MD software. However, PaCS-MD requires scripts for executing multiple MD simulations with different initial conditions, calculating the selection feature from the obtained trajectories, rank ordering of the snapshots based on the selection feature, and preparing the initial structures for the next cycle. We developed PaCS-Toolkit as an optimized package that facilitates PaCS-MD simulation and enables the use of different MD software and trajectory analysis tools. PaCS-Toolkit also aids in the analysis of the obtained trajectories including preparation for subsequent MSM analysis. PaCS-Toolkit includes tools for conducting a variety of PaCS-MD simulations. In this paper, we present the software design of PaCS-Toolkit and examples of its applications using the original PaCS-MD (t-PaCS-MD),^1^ dPaCS-MD,^4,5,11^ and rmsdPaCS-MD^13^ in combination with MSM.

## 2 Methods

### 2.1 Features of PaCS-Toolkit

PaCS-Toolkit is coded with Python and is compatible with Python 3.7 or later versions. PaCS-Toolkit requires standard Python libraries such as NumPy^47^ and SciPy^48^ and libraries for parallel processing. The user’s choice of MD software must be installed in advance. The current version, PaCS-Toolkit1.0, can employ GROMACS,^49,50^ AMBER,^51^ and NAMD.^52^ PaCS-Toolkit is compatible with various computing environments, including super computers with a message passing interface (MPI), servers with graphic processor units (GPUs), and personal computers such as laptops. PaCS-Toolkit heavily incorporates parallelization using MPI, GPU, and the multiprocessing package of Python, enabling optimization of the computation time depending on the available computational environments. PaCS-Toolkit allows easy customization by editing the configuration file and specifying the MD software to be used. PaCS-MD maintains flexibility so that new features, libraries, and software can be added by introducing responsible “classes” of Python.

The core of the PaCS-Toolkit for running PaCS-MD is composed of simulator, analyzer, and exporter. Simulator performs MD simulations via user-specified MD software. Analyzer analyzes MD trajectory files generated by simulator, calculates the selection feature, and ranks the snapshots. Exporter generates initial structures for the next cycle based on the rankings.

### 2.2 How to Use PaCS-Toolkit

Users can conduct PaCS-MD by preparing a single configuration file (*input.toml*) and necessary standard MD input files and by specifying options and I/O files. PaCS-Toolkit is executed by entering the command, “pacs [function] [parameters]”. Available functions are listed in Table 1. For example, PaCS-MD is performed by “pacs mdrun -t 1 -f *input.toml*”, wherein “mdrun” identifies the function to execute PaCS-MD, and “-t 1” indicates the number of PaCS-MD trials. The specified number is used as a part of the directory name, and “-f *input.toml*” specifies the configuration file.

**Table 1:** Functions of the PaCS-Toolkit

type of function	function	purpose
execution of PaCS-MD	mdrun	execute PaCS-MD
post-PaCS-MD tools	genrepresent	generate representative conformational pathways along the PaCS-MD cycles
	fit	regenerate MD trajectories after best-fitting selected regions/molecules to a reference
	gencom	generate the center of mass (COM) trajectories of a molecule in PDB format (.pdb)
	rmmol	reduce the size of MD trajectories by selecting necessary molecules and removing the rest
	rmfile	remove files unnecessary for PaCS-MD and analysis
MSM tool	genfeature	calculate the MSM features for MSM analysis and output them in NumPy format (.npy)

**Figure 2:**
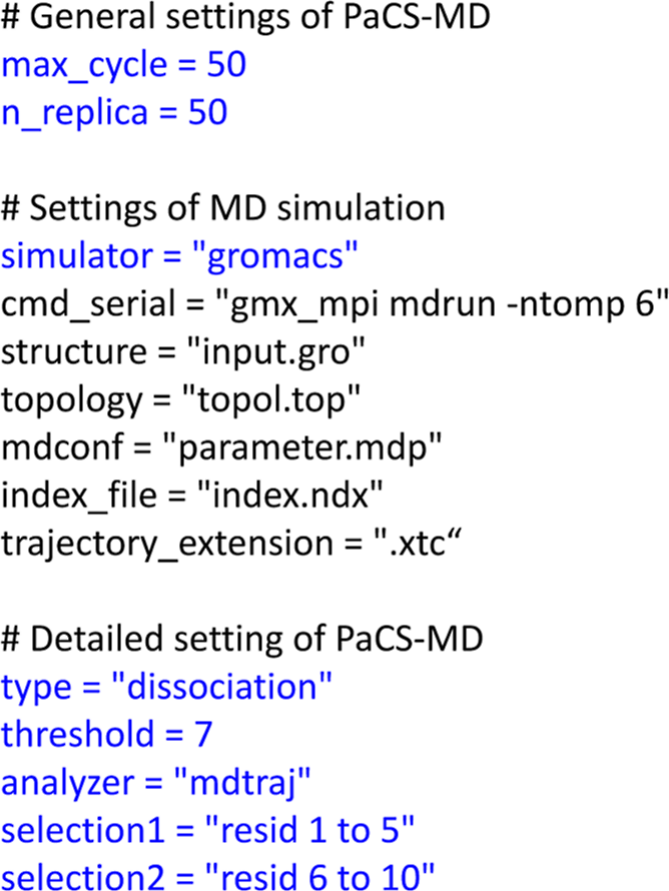
Example of the configuration file, *input.toml* for dPaCS-MD conducted with GROMACS. Major keywords explained in the text are shown in blue.

The content of *input.toml* controls the setting, I/O files, and use of modules with “keywords”. An example of the content of *input.toml* is shown in Figure 2. The choice of GROMACS,^49,50^ AMBER,^51^ or NAMD^52^ is indicated by the keyword, *simulator* = “gromacs”, “amber”, or “namd” in *input.toml*, respectively. The type of PaCS-MD to be conducted is determined by the keyword *type*, as shown in Table 2. User-defined selection features can be used by specifying *type* = “template” and by modifying the file *template.py*, in which three functions to calculate the selection feature must be described by the users. To analyze the MD trajectories, users should select from GROMACS,^49^ Cpptraj (the main program for processing trajectory files in AMBER),^53^ and MDtraj (Python libraries for MD trajectories).^54^ The choice is indicated by the keyword *analyzer* = “gmx”, “cpptraj”, or “mdtraj”, respectively.

**Table 2:** Available PaCS-MD Methods Are Specified by the Keyword *type*

types of PaCS-MD	specified value by *type*
t-PaCS-MD^1^	target
rmsdPaCS-MD^13^	rmsd
dPaCS-MD^4,5,11^	dissociation
aPaCS-MD^7^	association
a/dPaCS-MD^7^	a_d
eePaCS-MD^8,9^	ee
user defined feature	template

### 2.3 Typical Settings for the PaCS-MD

Important setting parameters for PaCS-MD include the number of replicas (*n*_rep_), the length of each MD simulation cycle (*t*_MD_), and the number of trials (*n*_trial_). PaCS-MD execution should be stopped when the number of cycles (*n*_cyc_) reaches a certain limit. In the configuration file, *n*_rep_ is specified by the keyword *n_replica*. In principle, the probability of the occurrence of stochastic events is proportional to *n*_rep_. The sampling efficiency therefore increases as a greater number of replicas is used, reducing the *n*_cyc_ required to reach a target.^4^ Typical *n*_rep_ values are 10–100. Users planning to construct an MSM from a single trial of PaCS-MD that generates samples along a narrow conformational transition pathway should use a minimum *n*_rep_ of 30 but 50 is better. When dPaCS-MD was conducted to sample dissociation pathways of a protein/peptide complex^19^ and protein/ligand complexes^11^ with *n*_rep_ = 10, 10–30 MD simulations per PaCS-MD cycle were additionally performed for some of the trials so that a one-dimensional MSM (1D-MSM) per pathway was constructed with sufficient statistics. These additional MD simulations were started from the initial structures of PaCS-MD cycles but with different initial velocities until sufficient samples were obtained to construct the MSM. Since the use of a larger *n*_rep_ reduces the *n*_cyc_ required to reach a target, PaCS-MD with *n*_rep_ = 30–50 will likely reduce the total computational cost compared to conducting PaCS-MD with *n*_rep_ = 10 and additional MD simulations later.

The length of MD simulation per cycle (*t*_MD_) is the MD time step Δ*t* multiplied by the number of MD iterations and should be specified inside the MD configuration file assigned by the keyword *mdconf*. One key factor in determining *t*_MD_ is the time scale of dynamics along the selection feature, so *t*_MD_ should be sufficiently long to observe fluctuation of the selection feature. Another key factor is the effect of the initialization protocol, which modulates the system only once per cycle. A shorter *t*_MD_ introduces a greater chance of modulation caused by initialization per fixed MD cost. A third key factor should be considered if the generated data are analyzed later by MSM to consider transitions between discrete microstates separated by a lag time τ.^55−58^ If *t*_MD_ is too short, it may be difficult to construct an MSM. For adequate statistics, τ should be the minimum of *t*_MD_/2 and the maximum of lag time before disconnecting the state involved in the slowest process.^59^ Thus, *t*_MD_ should be equal to or greater than 2τ, but the choice of τ also depends on the level of coarse-graining and discretization. The optimal value of *t*_MD_ should be determined based on the balance between these key factors. The choice of *t*_MD_ is strongly dependent on the dynamics of interest, which can vary from 0.1 ns to tens of nanoseconds or longer. In dPaCS-MD/MSM and a/dPaCS-MD/MSM, *t*_MD_ = 0.1 ns is frequently used, which is sufficient for constructing an MSM to calculate the free energy landscape and flux along the pathways and to reproduce experimentally determined Δ*G*°, and *k*_on_ and *k*_off_ values.^4,5,7,11,16,19^

The maximum limit of *n*_cyc_ is specified by the keyword *max_cycle*. *n*_cyc_ required to achieve the expected sampling strongly depends on the targets and typically varies from several to hundreds of cycles. The RMSD target value from a reference or *d*_com_ to be reached is specified with the keyword *threshold* (unit: nm). PaCS-MD stops when either *n*_cyc_ reaches *max_cycle*, or the condition indicated by *threshold* is satisfied, except for eePaCS-MD and a/dPaCS-MD, in which no threshold to interrupt the execution is specified. a/dPaCS-MD requires several different parameters.^7^ When *d*_com_ reaches the target value specified by the keyword *d_threshold* during the execution of dPaCS-MD, it switches to aPaCS-MD, and then when association movements stack for several cycles, the moving direction switches to dPaCS-MD. The switching condition is controlled by two keywords: *bound_threshold* defines the number of cycles wherein association movements stack before switching to dPaCS-MD; and *frame_sel* sets the number of first frames considered to judge the occurrence of stacking.

The residues and atoms involved the RMSD and *d*_com_ calculations are specified by the keywords, *selection1*, *selection2* (mandatory), *selection3*, and *selection4* (optional). In aPaCS-MD, dPaCS-MD, and a/dPaCS-MD, *selection1* and *selection2* specify the groups of residues and/or atoms (referred to as group1 and group2 hereafter) for which the centers of mass are calculated and *d*_com_ is determined between the two groups. To dissociate or associate two molecules, groups 1 and 2 should be selected from different molecules. The selection of two domains from the same protein allows interdomain motion to be simulated. In t-PaCS-MD and rmsdPaCS-MD, *selection1* indicates the residues and/or atoms of group1 to be best-fitted, and *selection2* defines the group2 whose coordinates are translated and rotated according to the best-fit performed with group1 and for which RMSD is calculated. Typically, group1 and group2 can be identical in t-PaCS-MD. The coordinate file of the reference structure for the RMSD calculation is indicated by the keyword, *reference*. The keywords *selection3* and *selection4* indicate the reference coordinates for groups 1 and group2, respectively. The specification of *selection3* and *selection4* is required only when the content of the reference file is somewhat different from the information in PaCS-MD. For example, if the original coordinate files of the Protein Data Bank are used as the reference, the residue numbering and contained atoms can be different from those in the MD software. When these two keywords are unspecified, they are automatically set to *selection1* and *selection2*, respectively. The formats to select residues/atoms depend on the choice of *analyzer* because the specification methods of the analyzer, gmx, cppdtraj, and mdtraj, are used.

The number of trials (*n*_trial_) required for adequate sampling depends on the purpose of the simulation. *n*_trial_ should be determined so that the conformational space is sufficiently sampled to calculate the target quantities. The minimum *n*_trial_ necessary to calculate the binding free energies of complexes with dPaCS-MD/MSM is around five^4,11,19^ because the free energy difference between a well-defined bound state and sufficiently distant unbound states should in principle be path independent and can be calculated without extensive sampling of many different pathways. However, at least 10 or more trials are required to sample significant pathways covering different directions when path-dependent dissociation mechanisms are investigated^16^ or experimentally measured *k*_on_ and *k*_off_ are to be reproduced.^5^ As mentioned previously, the trial number is specified by the “-t” option when PaCS-MD is executed with “pacs mdrun”.

### 2.4 Applications

Three different types of simulations conducted with the PaCS-Toolkit are demonstrated in this paper. All the MD and PaCS-MD simulations were performed by using GROMACS^49,50^ with a Nosé–Hoover thermostat^60,61^ and Parrinello–Rahman barostat^62^ employed to achieve isothermal and isobaric conditions, respectively. GROMACS was used as the analyzer. The “genfeature” function of PaCS-Toolkit (Table 1) was used to output the MSM features and pyEMMA2^63^ was employed to construct MSM. *k*-means clustering was performed with the *k*++ algorithm^64^ to discretize the MSM features into microstates.

#### 2.4.1 Folding of the “Mini-Protein” Chignolin by t-PaCS-MD/MSM

Chignolin is a designed peptide consisting of 10 amino acid residues (GYDPETGTWG) and is a “mini-protein” that folds into a β-hairpin structure (PDB ID: 1UAO).^65^ Here, chignolin was first simulated at 500 K so that it completely unfolded; then, using t-PaCS-MD started from considerably different structures, it was folded into a native fold.

The initial structure of chignolin taken from model 1 of the 1UAO PDB file^65^ was solvated into a 5.4 × 5.4 × 5.4 nm^3^ water box with 0.15 M NaCl comprising 11,512 atoms in total. The CHARMM36m and TIP3P(CHARMM) force fields^66^ were used for chignolin and the water molecules, respectively. After energy minimizations with the steepest descent method followed by the conjugate gradient method, MD simulation was conducted at 500 K for 1 ns with restraints to maintain native hydrogen bonds with the *NVT* ensemble and then continued for 50 ns at 500 K and 1 bar with the *NPT* ensemble. The MD time step was 1 fs in all of the simulations. The following 200 ns MD simulation was performed without restraints to sample significantly different unfolded structures, and four initial structures for t-PaCS-MD were randomly selected from representative structures of clustered chignolin snapshots.

t-PaCS-MD was conducted 20 times (5 times per initial structure with different initial velocities) with *n*_rep_ = 50, *t*_MD_ = 0.1 ns, *max_cycle* = 30, and *threshold* = 0.05 nm, employing the backbone RMSD from model 1 of the 1UAO PDB file^65^ as the selection feature. Although the RMSD threshold of 0.1 nm is sufficient to reach around the native state, the value of 0.05 nm was chosen so that conformations around the native state are sufficiently sampled. The actual PaCS-MD cycles finished within *n*_cyc_ ≤ 30 in eight cases. Seventeen trials out of 20 satisfied the condition RMSD <0.1 nm within 30 cycles. To obtain more samples around the native state, additional 2 ns MD simulations were performed starting from the last snapshots of these trials.

We constructed the MSM by employing the three hydrogen bond distances, HB1, HB2, and HB3 as the MSM features. HB1 is formed between the main chain amide N atom of Asp3 (hydrogen bond donor) and the main chain carbonyl O atom of Thr8 (acceptor). HB2 is an alternative to HB1 formed between Asp3:N and Gly7:O. HB1 and HB2 are considered good indicators to distinguish the native (HB1) and misfolded (HB2) states.^1,67−70^ HB3 (Gly7:N–Asp3:O) is predominantly formed before HB1 and HB2 are formed.^1^ The trajectories in 3D space spanned by the distances of HB1, HB2, and HB3 were clustered into 2000. The trajectories with the HB3 distance >4 Å were excluded in this step, similar to the methods used in refs,^1,67,68^ 3D-MSM was conducted with the lag time of 50 ps, and the free energy landscape of the folding pathways was calculated. The implied time scale versus lag time plot for the MSM analysis is shown in Figure S1.

#### 2.4.2 Domain Motion of the SARS-CoV-2 Nsp15 Monomer by rmsdPaCS-MD/MSM

The Nsp15 protein of coronavirus cleaves the polyuridine (polyU) of negative-sense RNAs, limits the abundance and length of polyU, and delays the type I interferon response in macrophages.^71^ The crystal structure of the SARS-CoV-2 Nsp15 hexamer in its apo state (PDB ID: 6VWW) reveals a distinctive ring-like complex comprising a dimer of trimers.^72^ Each monomer structure is characterized by three distinct domains: the N-terminal domain, spanning residues 1 to 68 (called the N-term domain hereafter); the middle domain, encompassing residues 69 to 202 (Mid domain); and the C-terminal domain that contains the NendoU catalytic site and spans residues 203 to 347 (C-term domain). Although the hexamer structure suppresses domain movements, the Nsp15 monomer was shown to be very flexible and could potentially be a target of anti-SARS-CoV-2 drugs.^13^

The simulated system contained the Nsp15 monomer solvated in a 15.9 × 14.7 × 14.7 nm^3^ box filled with water molecules and 0.15 M KCl. The AMBER ff19SB force field^73^ was used for the protein and the OPC water model^74^ was employed. The equilibrated monomer structures of Nsp15 at 300 K and 1 atm obtained in an earlier paper^13^ were employed as the initial structures for rmsdPaCS-MD.

Using backbone RMSD from the crystal monomer structure as the selection feature and selecting snapshots with greater RMSD, rmsdPaCS-MD at 300 K and 1 bar was conducted 20 times with *n*_rep_ = 50 and *t*_MD_ = 0.1 ns. In all of the trials, *n*_cyc_ reached *max_cycle* = 50 because *threshold* was set to a very large value (10 nm).

1D-MSM was conducted using *d*_com_ between the N- and C-term domains as the MSM feature with a lag time of 50 ps, and the free energy profile of the domain motion as a function of *d*_com_ was calculated. The implied time scale versus lag time plot for the MSM analysis is shown in Figure S2.

#### 2.4.3 Ligand Dissociation from the Adenosine A_2A_ Receptor by dPaCS-MD/MSM

Adenosine A_2A_ receptor (A_2A_R) is a member of the G protein-coupled receptor (GPCR) superfamily, plays central roles in sleep regulation, angiogenesis, and immunosuppression, and is considered an important drug target.^75^ LUF5833 is a nonribose partial agonist of A_2A_R that binds in the binding pocket.^76^ The dissociation of LUF5833 from A_2A_R was simulated by dPaCS-MD. MD simulation of the A_2A_R/LUF5833 complex was conducted as follows to prepare initial structures for dPaCS-MD. The crystal structure of the A_2A_R/LUF5833 complex (PDB ID: 7ARO) was embedded in a membrane consisting of 145 POPC (1-palmitoyl-2-oleoyl-*sn*-glycero-3-phosphocholine) and 37 cholesterol molecules and was solvated in water with 0.15 M KCl in a 7.7 × 7.7 × 16.6 nm^3^ box using CHARMM-GUI.^77^ The system comprised 125,557 atoms. AMBER ff19SB,^73^ Lipid21,^78^ and OPC^74^ were employed for the protein, membrane, and water molecules, respectively. Partial charges of LUF5833 were generated by GAFF2^79^ using Gaussian16^80^ with B3LYP/6-31G* and Antechamber in AmberTools.^81^

After energy minimizations with the steepest descent method followed by the conjugate gradient method, MD simulation was conducted for 1 ns at 300 K with the *NVT* ensemble and continued for 100 ns at 300 K and 1 bar with the *NPT* ensemble with positional restraints imposed on the A_2A_R/LUF5833 heavy atoms. The MD time step was 2 fs. Ten independent MD simulations were then conducted without restraints for 1 μs. Snapshots of the second halves of the trajectories were gathered and clustered into 10 groups, and a representative structure was selected from each cluster as the initial structure for the dPaCS-MD. Therefore, ten initial structures were selected for the following PaCS-MD.

*d*_com_ between the two molecules was used to dissociate LUF5833 from A_2A_R as the selection feature and dPaCS-MD was conducted 30 times (3 times per initial structure with different initial velocities) with *n*_rep_ = 50, *t*_MD_ = 0.1 ns, and an MD time step of 1 fs. The simulations were conducted with *max_cycle* = 100 with an extremely large *threshold* value (100 nm), and the distance exceeded 6.0 nm within the designated cycles in all trials. *k*-Means clustering into 50 was conducted for the MSM analysis using snapshots with *d*_com_ ≤ 6 nm, and a lag time of 50 ps was employed to construct 1D-MSM using *d*_com_ as the MSM feature. The implied time scale versus lag time plot for the MSM analysis is shown in Figure S3. Δ*G*° was calculated from the potential of mean force obtained by the MSM with volume correction^82,83^ conducted with the procedure described elsewhere^11^ by volume calculation using the convex hull with the Quickhull algorithm^21^ implemented in SciPy.^48^

## 3 Results and Discussion

### 3.1 Folding of Chignolin

**Figure 3:**
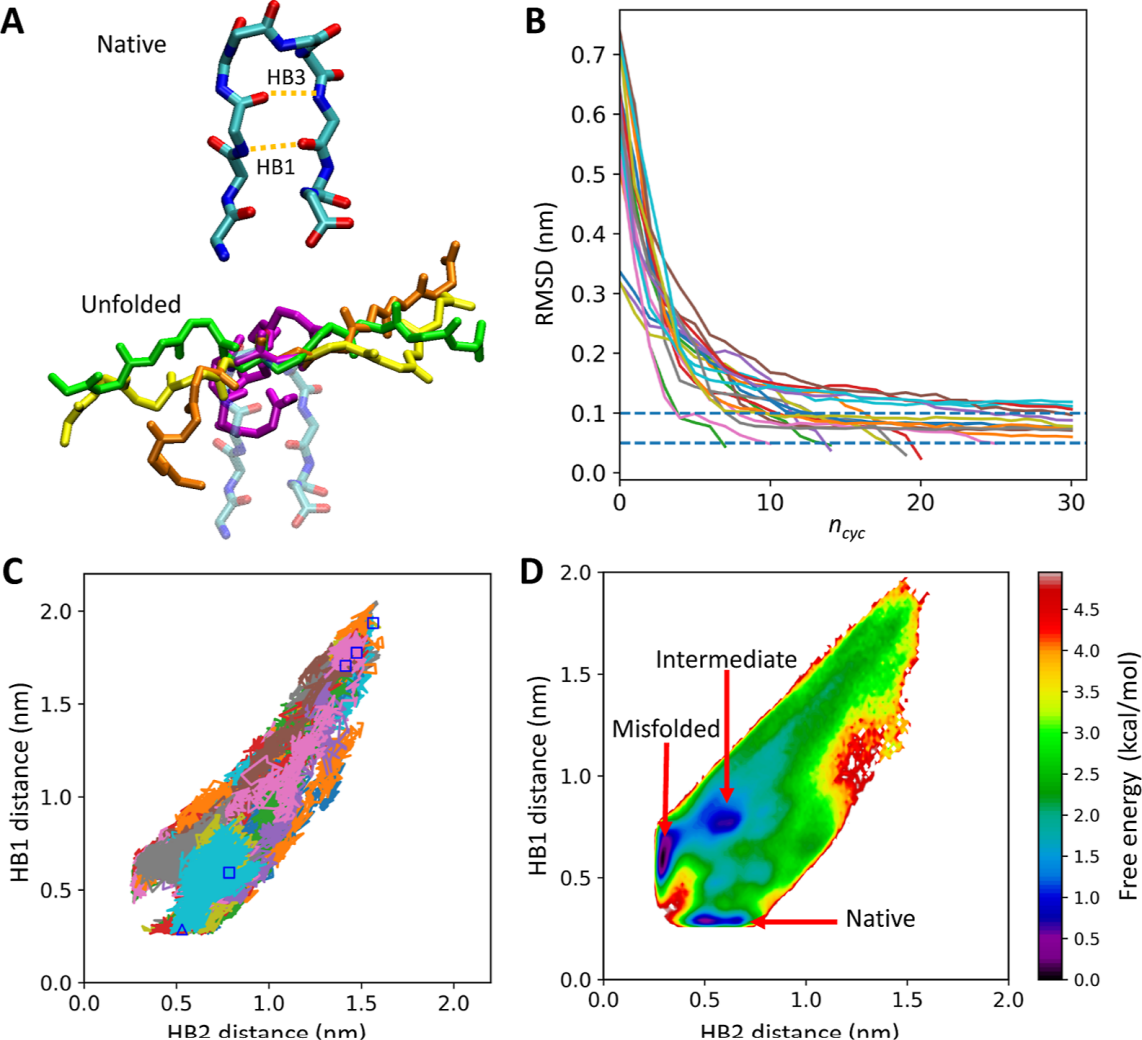
Results of t-PaCS-MD/MSM on chignolin. (A) The native structure of chignolin (the top image. model 1 of 1UAO(65)) and four unfolded starting structures for t-PaCS-MD (the bottom image. green, yellow, orange, and magenta) best-fitted to residues 5 and 6 of the native structure are shown transparently. The images in Figures 1–3 were created by VMD^84^ unless otherwise specified. (B) Backbone RMSD (the selection feature) as a function of *n*_cyc_ during t-PaCS-MD. The horizontal lines show 0.1 and 0.05 nm. (C) Projections of folding pathways mapped onto the space spanned by the distances of HB1 and HB2. Representative pathways are selected from each trial and are shown in different colors. The square symbols indicate the positions of the initial structures, and the open triangle represents the native structure. (D) The free energy landscape of chignolin folding mapped onto the HB1–HB2 distance space.

Folding simulation of chignolin by t-PaCS-MD was started from four different structures generated by the MD simulation. The initial structures and native structure of chignolin are shown in Figure 3A. In 17 trials out of 20, chignolin folded into the native structure (RMSD < 0.1 nm) within 30 cycles as indicated by the plot of RMSD versus *n*_cyc_ (Figure 3B). Figure 3C shows representative folding pathways of chignolin generated by t-PaCS-MD. Most of the pathways led to the native state directly, but some passed through the misfolded state in which HB2 formed instead of HB1. Figure 3D represents the free energy landscape on the space spanned by the distances of HB1 and HB2, showing free energy minima of the native, misfolded, and intermediate states.^1,67,68^

### 3.2 Domain Motion of Nsp15

**Figure 4:**
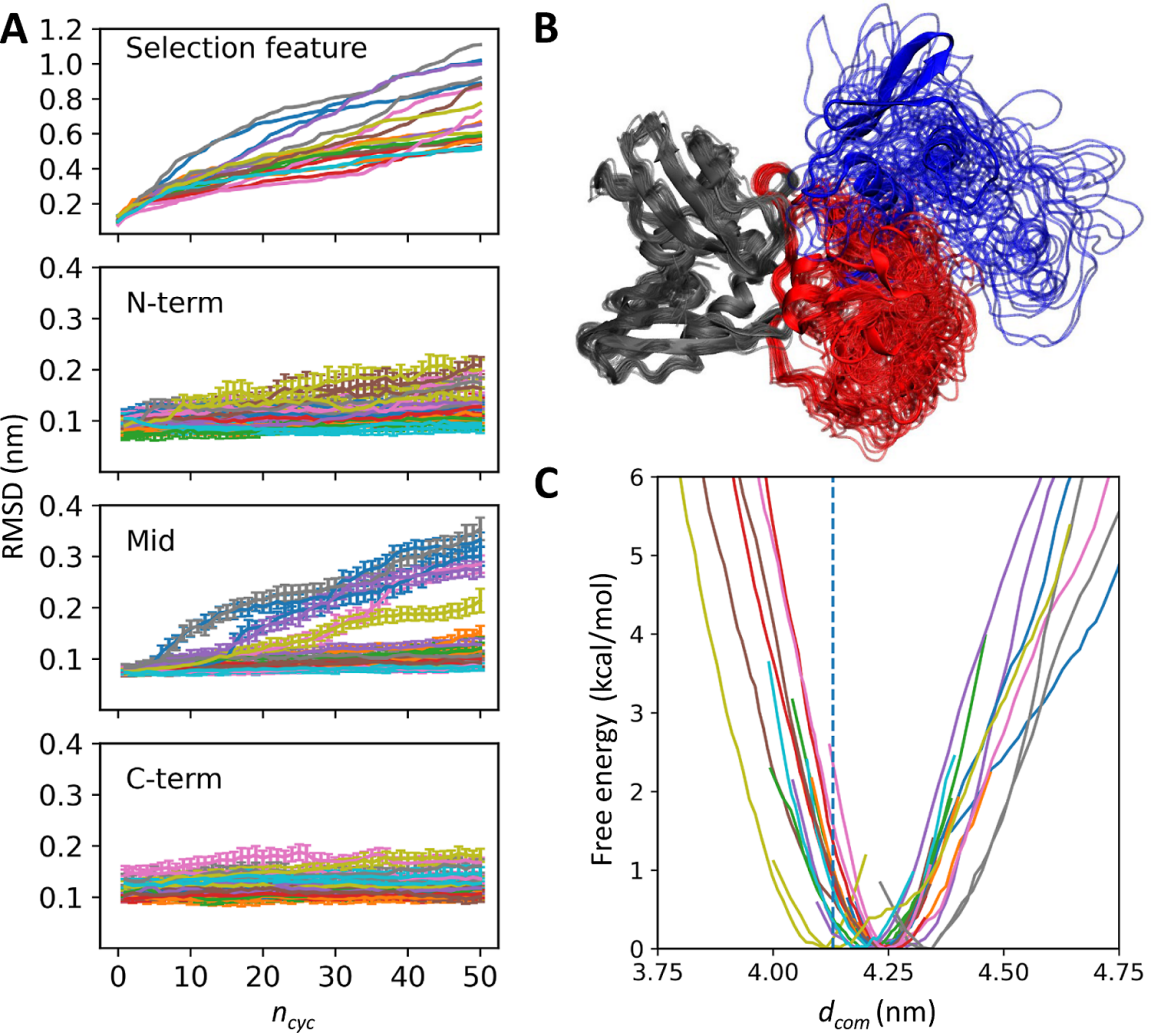
Results of rmsdPaCS-MD/MSM on the Nsp15 monomer. (A) Backbone RMSD as a function of *n*_cyc_ during rmsdPaCS-MD for each trial. RMSDs for the whole molecule (the selection feature) and the N-term (blue), Mid (red), and C-domains (gray) are shown. The error bars represent the standard deviations between the replicas. (B) The monomer structure in the apo hexamer^72^ (cartoon representation) and examples of conformations generated by the rmsdPaCS-MD (thin tube representations) after best-fitting the C-term domain. The last snapshots of all of the trials from the first replica at *n*_cyc_ = 50 are shown. (C) The free energy profile as a function of *d*_com_ between the N- and C-term domains. The vertical line shows the *d*_com_ of the apo hexamer.

The domain motion of Nsp15 was significantly enhanced by rmsdPaCS-MD. The backbone RMSD for the whole molecule was around 0.5–1.1 nm, indicating that large movements were enhanced by rmsdPaCS-MD (Figure 4A). RMSDs for each domain were around 0.1–0.2 nm except for Mid domain in six trials where RMSDs were greater than 0.2 nm. These results show that the domain structures are mostly well maintained, and rmsdPaCS-MD enhanced domain motions, as shown in Figure 4B, and especially large movements of the N-term domain relative to the other domains. The data used for constructing Figure 4B were prepared by the PaCS-Toolkit function “genrepresent” (see Table 1). Figure 4C shows the free energy profile calculated by 1D-MSM as a function of *d*_com_ between the N- and C-term domains, showing that the Nsp15 monomer adopts a more open form compared to the apo hexamer structure, consistent with an earlier study.^13^

### 3.3 Ligand Dissociation from A_2A_R

**Figure 5:**
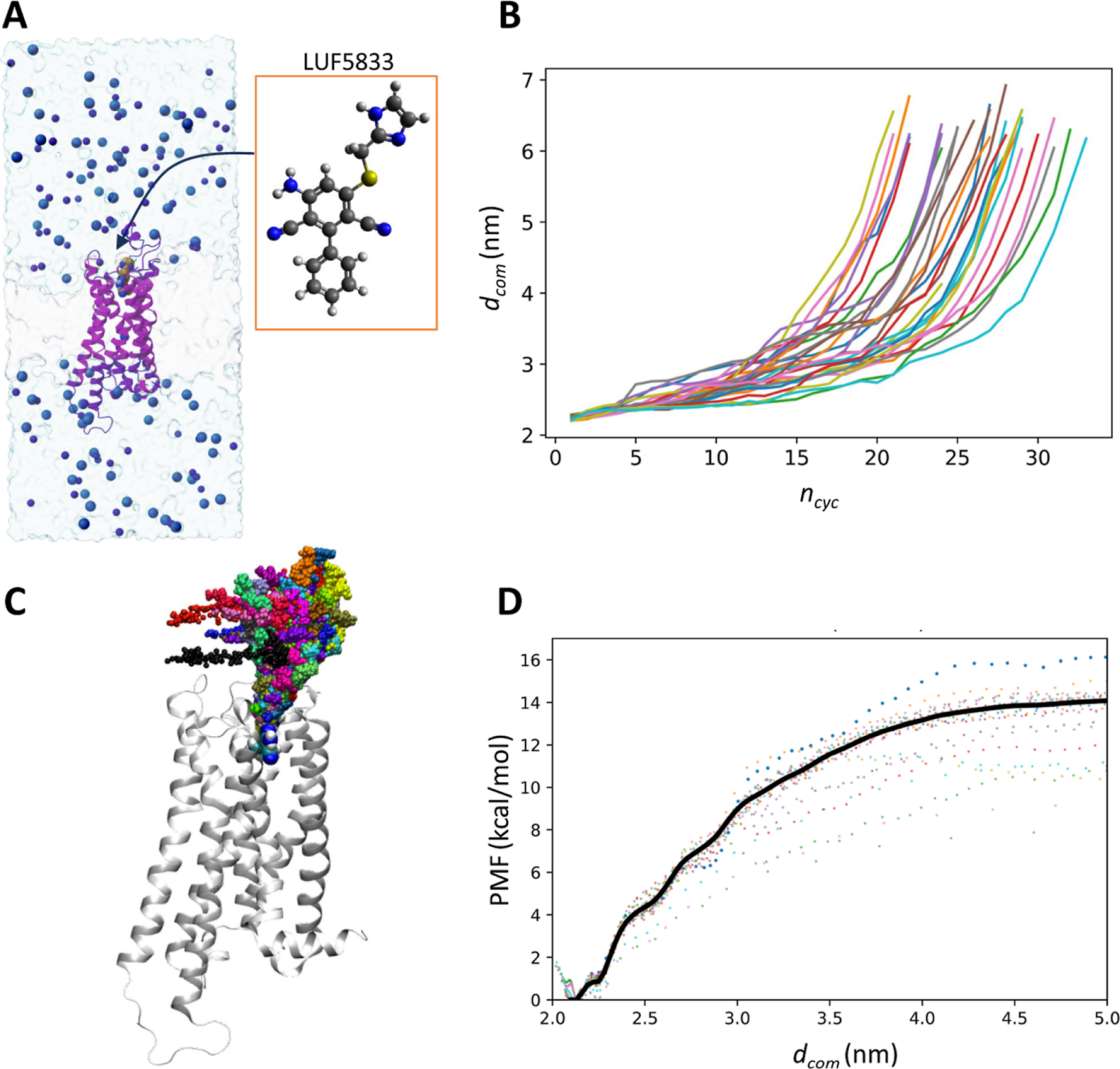
Results of dPaCS-MD/MSM on the A_2A_R/LUF5833 complex. (A) A side view of the simulated system containing A_2A_R (magenta), LUF5833 (spheres), the membrane region (white), water (blue), K^+^ (purple spheres), and Cl^–^ (cyan spheres). The image was created using ChimeraX.^86−88^ The chemical structure of LUF5833 is shown in the inset. (B) The intercenter-of-mass distance *d*_com_ (the selection feature) as a function of *n*_cyc_ during dPaCS-MD. (C) The dissociation pathways of LUF5833 from A_2A_R (white cartoon model). The center of mass positions of LUF5833 along the dissociation pathways are shown as spheres in path-dependent colors. (D) The potential of mean force (PMF) as a function of *d*_com_. The thick black line represents the average PMF and the dotted lines show individual PMFs from each trial.

Dissociation of LUF5833 from A_2A_R was investigated by dPaCS-MD/MSM. Figure 5A shows the simulated system containing the A_2A_R/LUF5833 complex. The plot of the selection feature *d*_com_ versus *n*_cyc_ (Figure 5B) indicates that, in all 30 trials, LUF5833 dissociated up to 6 nm or more from A_2A_R within 30 cycles, with only up to 3 ns (10^–9^ s) required for each dPaCS-MD trial. The experimental value of *k*_off_ is 0.16 ± 0.08 min^–1^, meaning that dissociation takes the order of 1 × 10^2^ s. Therefore, the ratio of acceleration is on the order of 1 × 10^11^. Figure 5C depicts the center of mass positions of LUF5833 along the dissociation pathways sampled by dPaCS-MD, showing that after exiting from the narrow binding pocket of A_2A_R, LUF5833 diffused in different directions. The presented data were prepared by the PaCS-Toolkit function “gencom” (see Table 1). Figure 5D shows the potential of mean force (PMF) calculated by 1D-MSM as a function of *d*_com_, which mostly converges between 4.5 and 5.0 nm. The value of Δ*G*° deduced from the PMF after volume correction^82,83^ is −10.9 ± 0.2 kcal/mol. Kinetic *K*_D_ obtained as experimentally measured *k*_off_/*k*_on_ for LUF5833 is 19 ± 0.6 nM,^85^ which is equivalent to Δ*G*° = −10.6 kcal/mol. The Δ*G*° value deduced from dPaCS-MD/MSM is in good agreement with the experimental value.

## 4 Conclusions

In this paper, we presented the software design of the PaCS-Toolkit. PaCS-Toolkit is coded with Python3 and is distributed under the GPLv3 license. PaCS-Toolkit, its documents, and input files for the examples presented in this paper are available from GitHub.^a^ Potential users can download the PaCS-Toolkit and input examples and start to conduct PaCS-MD with the provided inputs. The users who can program with Python should be able to modify the code of the PaCS-Toolkit or implement new methods.

We also demonstrated three different types of applications. The folding of chignolin investigated by t-PaCS-MD/MSM identified direct folding into the native state as well as folding via a misfolded state, similar to the results of previous studies.^1,67,68^ rmsdPaCS-MD/MSM of the Nsp15 monomer successfully enhanced domain motions without causing large intradomain motion in the majority of cases and identified that the open structure is stable in the monomer state compared to the more closed structure in the hexamer form. The dissociation of LUF5833 from A_2A_R was successfully simulated within 3 ns of dPaCS-MD and the experimental value of Δ*G*° was well reproduced by dPaCS-MD/MSM. These results indicated that PaCS-Toolkit can be easily utilized to simulate a wide variety of dynamics for different types of molecular systems. As mentioned earlier, input files for these examples are available.

Since the PaCS-Toolkit is designed to be flexible, new features can be incorporated relatively easily. For example, although PaCS-Toolkit1.0 supports only GROMACS, AMBER, and NAMD, other MD software can be made available. Also, variations of PaCS-MD not implemented in the current version and new types of PaCS-MD can be integrated in the future.

## Supporting Information Available

Implied time scale versus lag time plots for the MSM analysis for chignolin folding, dynamics of the Nsp15 monomer, and LUF5833 dissociation from A_2A_R (PDF)

## Footnotes

a PaCS-Toolkit is available from GitHub https://github.com/Kitaolab/PaCS-Toolkit under the GPLv3 and examples of input files for the three applications shown in this paper are available from GitHub https://github.com/Kitaolab/PaCS-MD-example.
