# Supplementary material for: PaCS-Toolkit: Optimized Software Utilities for Parallel Cascade Selection Molecular Dynamics (PaCS-MD) Simulations and Subsequent Analyses
Source: J Phys Chem B. 2024 Apr 5;128(15):3631–42. doi: 10.1021/acs.jpcb.4c01271 (PMC11033871; doi:10.1021/acs.jpcb.4c01271)

Supporting Information for:

PaCS-Toolkit: Optimized Software Utilities for Parallel Cascade Selection Molecular Dynamics (PaCS-MD) Simulations and Subsequent Analyses.

Shinji Ikizawa[1,‡], Tatsuki Hori[1,‡], Tegar Nurwahyu Wijaya[1,2,‡], Hiroshi Kono[1], Zhen Bai[1], Tatsuhiro Kimizono[1], Wenbo Lu[1], Duy Phuoc Tran[1], Akio Kitao[1,]*

[1] School of Life Science and Technology, Tokyo Institute of Technology, 2-12-2 Ookayama, Meguro, Tokyo 152-8550, Japan

[2] Department of Chemistry, Universitas Pertamina, Jl. Teuku Nyak Arief, Simprug, Jakarta 12220, Indonesia

‡These authors contributed equally.

*Corresponding author: Akio Kitao, School of Life Science and Technology, Tokyo Institute of Technology, M6-13, 2-12-1 Ookayama, Meguro-ku, Tokyo 152-8550, Japan

E-mail: akitao@bio.titech.ac.jp

Figure S1. Implied timescale versus lag time plot for the MSM analysis of the chignolin folding.

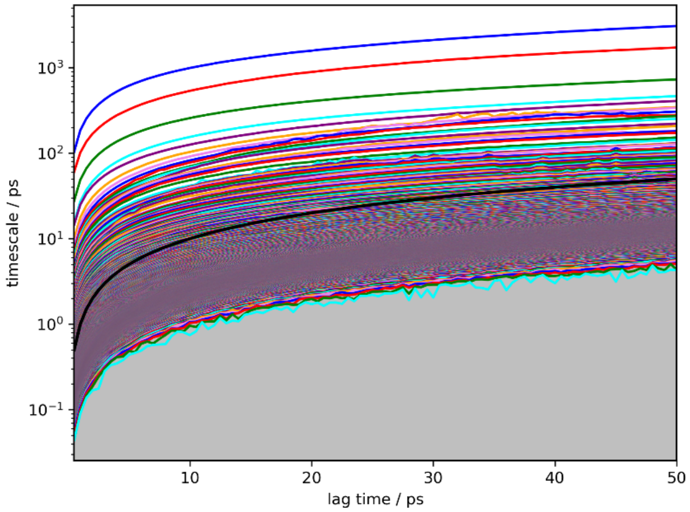

Figure S2. Implied timescale versus lag time plots for the 1D-MSM analysis of the dynamics of the Nsp15 monomer. The results of 20 trials are shown.

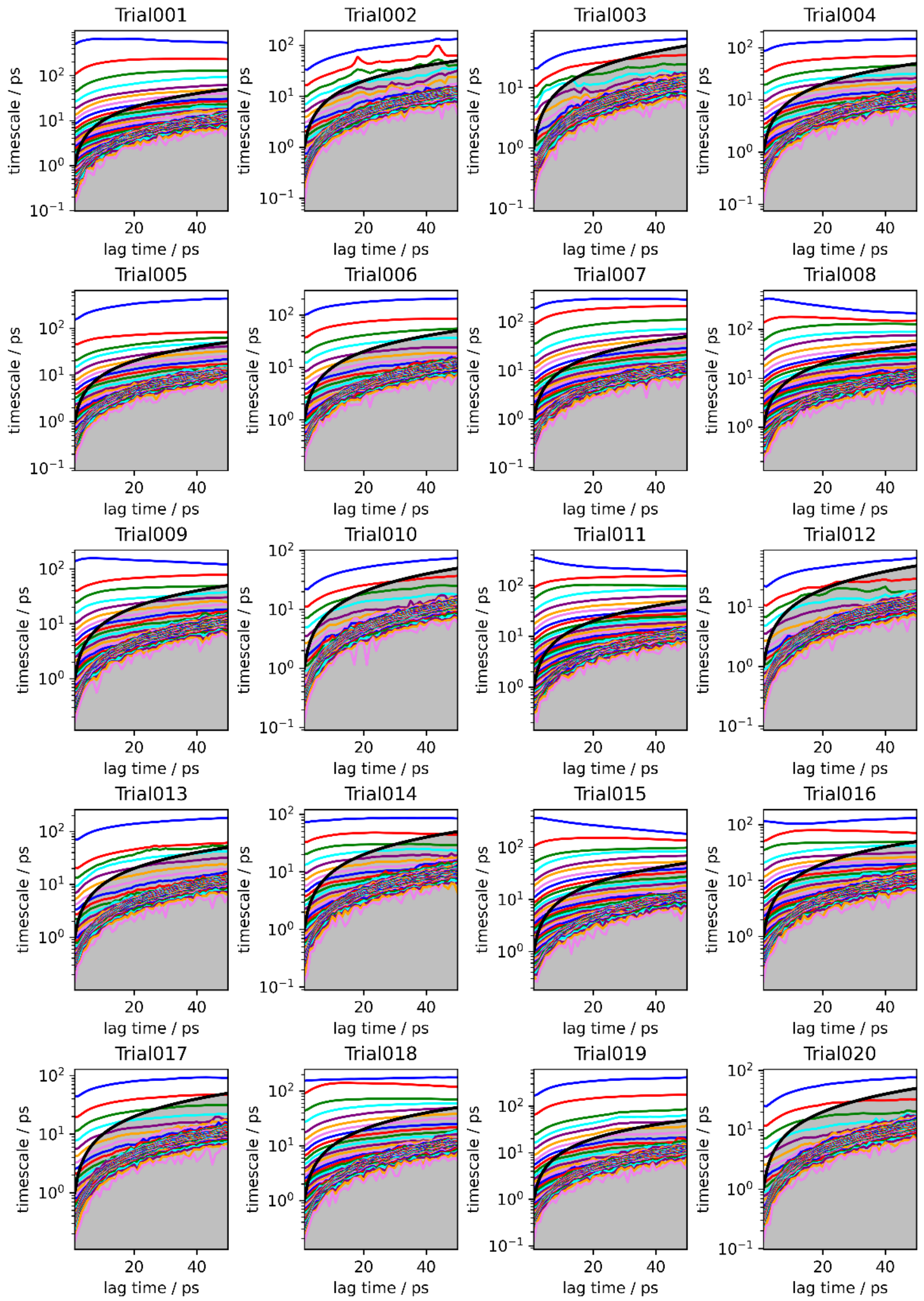

Figure S3. Implied timescale (ITS) versus lag time plots for the 1D-MSM analysis of LUF5833 dissociation from $A_{2A}R$. The results of 30 trials are shown.

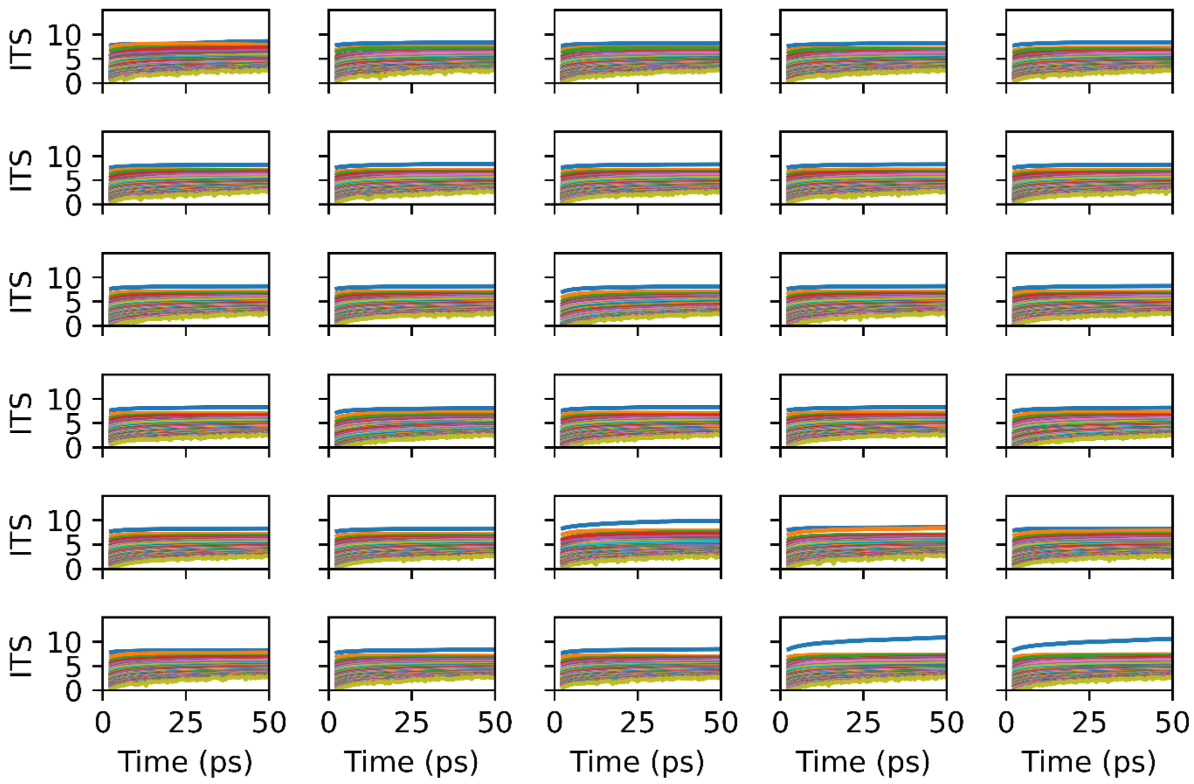

Supplement: Supplementary file 1 — jp4c01271_si_001.pdf [file jp4c01271_si_001.pdf]
